# Factors influencing on-scene time in a physician-staffed helicopter emergency medical service (HEMS): a retrospective observational study

**DOI:** 10.1186/s13049-023-01085-x

**Published:** 2023-04-14

**Authors:** Alexander Fuchs, Markus Huber, Thomas Riva, Stefan Becker, Roland Albrecht, Robert Greif, Urs Pietsch

**Affiliations:** 1 Department of Anaesthesiology and Pain Medicine, Inselspital, Bern University Hospital, University of Bern, Freiburgstrasse, 3010 Bern, Switzerland; 2 Swiss Air-Rescue (Rega), Zurich, Switzerland; 3 Department of Anaesthesiology and Intensive Care Medicine, Cantonal Hospital St. Gallen, St. Gallen, Switzerland; 4 University of Bern, Bern, Switzerland; 5 School of Medicine, Sigmund Freud University Vienna, Vienna, Austria; 6 European Resuscitation Council (ERC) Research NET, Niel, Belgium; 7 Department of Emergency Medicine, Inselspital, Bern University Hospital, University of Bern, Bern, Switzerland

**Keywords:** Helicopter emergency medical system, Prehospital on-scene time, Trauma, Helicopter hoist operation, Airway management, Resuscitation, Paediatric

## Abstract

**Background:**

For helicopter emergency service systems (HEMS), the prehospital time consists of response time, on-scene time and transport time. Little is known about the factors that influence on-scene time or about differences between adult and paediatric missions in a physician-staffed HEMS.

**Methods:**

We analysed the HEMS electronic database of Swiss Air-Rescue from 01-01-2011 to 31-12-2021 (N = 110,331). We included primary missions and excluded missions with National Advisory Committee for Aeronautics score (NACA) score 0 or 7, resulting in 68,333 missions for analysis. The primary endpoint ‘on-scene time’ was defined as first physical contact with the patient until take-off to the hospital. A multivariable linear regression model was computed to examine the association of diagnosis, type and number of interventions and monitoring, and patient's characteristics with the primary endpoint.

**Results:**

The prehospital time and on-scene time of the missions studied were, respectively, 50.6 [IQR: 41.0–62.0] minutes and 21.0 [IQR: 15.0–28.6] minutes. Helicopter hoist operations, resuscitation, airway management, critical interventions, remote location, night-time, and paediatric patients were associated with longer on-scene times.

**Conclusions:**

Compared to adult patients, the adjusted on-scene time for paediatric patients was longer. Besides the strong impact of a helicopter hoist operation on on-scene time, the dominant factors contributing to on-scene time are the type and number of interventions and monitoring: improving individual interventions or performing them in parallel may offer great potential for reducing on-scene time. However, multiple clinical interventions and monitoring interact and are not single interventions. Compared to the impact of interventions, non-modifiable factors, such as NACA score, type of diagnosis and age, make only a minor contribution to overall on-scene time.

## Introduction

Critically ill or injured patients require a rapid assessment and state-of-the-art medical treatment [1]. Furthermore, safe and fast transport to an appropriate hospital is necessary to prevent the patient from undergoing an avoidable secondary inter-hospital transfer that consumes more time [2, 3]. In Switzerland, the emergency medical system consists of paramedic-staffed ground ambulances and the emergency, physician-staffed Helicopter Emergency Medical Services (HEMS). HEMS patient transport is considered safe, ensures short intervention times and offers access to difficult and remote regions [4–6]. On-scene HEMS physicians diagnose patient illnesses or conditions earlier and perform or initiate advanced life-saving critical interventions, which improve the survival rates of trauma patients [6, 7].

Current international treatment guidelines for critical illnesses or injuries focus on the first critical hour. However, the association between prehospital time and survival is unclear [8]. Prehospital time consists of three parts: (1) the response time, including the flight time from the base to the patient; (2) the on-scene time; and (3) the time needed to transport the patient to the hospital. Prolonged on-scene time is associated with increased in-hospital mortality in trauma patients [9, 10]. By contrast, HEMS transport is an independent factor for improved survival in paediatric and adult trauma patients [11–13]. Commencing definitive treatment more rapidly contributes to improved survival, including for non-trauma patients, especially in stroke, myocardial infarction, cardiac arrest and sepsis [14–16].

The factors contributing to a mission’s prehospital time in a physician-staffed HEMS are unclear. Flight times depend not only on the distance from the base to the scene and the destination hospital, but also on the topography and weather conditions. As regards flight safety, these variables are unchangeable. Thus, we aimed to investigate factors that contribute to extending on-scene time. Furthermore, we were interested in the differences between paediatric and adult patients.

## Methods

### Study design and population

The study protocol of this retrospective observational cohort study was approved by the Ethics Committee of Eastern Switzerland (EKOS 22/021, St. Gallen, Switzerland), which waived the need for informed consent due to the retrospective study design and anonymised data analysis. The study was performed in line with the Declaration of Helsinki and the Swiss Act on Human Research. We followed the guidelines on Strengthening the Reporting of Observational Studies in Epidemiology (STROBE) [17].

### Setting

Swiss Air-Rescue (Rega) provides 24/7 physician-staffed HEMS services with 20 helicopters at 14 bases covering an area of more than 41,000 km^2^. The bases are distributed throughout the country, making it possible to reach any location within an average of 15 min after an alert is received. Swiss Air-Rescue operates a national dispatch and mission-control centre for HEMS operations and, independently of the government and hospitals, conducts around 14,000 HEMS missions annually. An HEMS crew consists of a pilot, a paramedic and a physician. Swiss Air-Rescue’s HEMS physicians require board certification in anaesthesiology and certification in prehospital emergency medicine.

### Data collection

We retrospectively screened the Swiss Air-Rescue HEMS electronic medical record database from 01-01-2011 to 31-12-2021 and included all primary HEMS missions. We excluded secondary missions, missions with National Advisory Committee on Aeronautics (NACA) score 0 and 7 (uninjured or dead patients) and missions lasting longer than 24 h (search and rescue missions). Patients under the age of 16 years were classified as paediatric, all others as adults. On-scene time, the primary endpoint, was defined as the landing time of the helicopter as a surrogate for the first physical contact between the crew and the patient until the beginning of the transport phase to the hospital with the lift-off of the helicopter. Secondary endpoints were: prehospital time, trauma vs. non-trauma and interventions or measurements influencing on-scene time.

### Measurements

The anonymised data was transferred from Swiss Air-Rescue’s electronic medical records into a dedicated research database. Patient and mission characteristics included age, sex, location (urban vs. remote), time of day and type of activity (e.g. traffic, sport, outdoor activity). The patients' location was defined as urban when access by road was given; otherwise remote. The medical data included the NACA score, Glasgow Coma Scale (GCS), and helicopter hoist operation. Diagnoses were coded according to the 2019 World Health Organisation International Classification of Diseases (ICD-10) and grouped into trauma and non-trauma [18]. Performance of medical interventions or monitoring by the HEMS crew are listed in Table 1 and were grouped into clinically meaningful sub-units for the multivariable model.

**Table 1:** Intervention and monitoring performed by the helicopter emergency medical system crew listed by sub-units

Basic interventions and monitoring	Immobilisation and analgesia	Critical interventions	Airway management	Resuscitation	Other
Intravenous (i.v.) access	Vacuum mattress	Intraosseous (i.o.) access	Tracheal intubation	Chest compressions	Cervical collar
Peripheral oxygen saturation (SpO_2_)	Sedation	Emergency front of neck access (eFONA)	Facemask ventilation	Defibrillation	Vacuum splinting
Electrocardiogram (ECG)	Analgesia	Chest needle decompression	Neuromuscular blocking agent (NMBA)	Mechanical chest compression device	Active rewarming (hot pads)
Temperature	Reduction of a fracture or dislocation	Transcutaneous paceing	Capnography		Invasive or non-invasive blood pressure
	Haemostasis	Vasopressors			Medication (Antiepileptic, Antiemetic, Tranexam acid, Antiarrhythmic, Antihypertensive, etc.)

For pragmatic medical reasons—and deviating from the formal definition of night flights—daytime missions were defined as those occurring between 07:00 and 19:00, while night time flights between 19:01 and 06:59. Mission times were defined as follows:

- On-scene time: from first physical contact with the patient until beginning of the flight to the destination hospital.
- Prehospital time: from the alerting of Swiss Air-Rescue’s national HEMS dispatch and mission-control centre until arrival at the destination hospital.

### Statistical analysis

In terms of summary measures, categorical variables were summarised using counts and percentages, while numerical variables were summarised using the median and interquartile range (IQR). Data availability is shown for each variable in the corresponding tables. We used chi-squared or exact Fisher test for unadjusted group comparisons between child and adult patients of categorical variables, and unpaired two-sample Wilcoxon test for numerical variables.

For the multivariable linear regression model with the outcome ‘on-scene time’, we included all patients with available measurements and a diagnosis, but excluded missions with pre-hospital times greater than 24 h. Model fit was evaluated by means of the adjusted R^2^. The effect of each variable on the outcome ‘on-scene time’ is illustrated by estimated marginal means. Separate multivariable linear regression models were computed for the child and adult sub-groups.

Due to the observational character of the study, no formal sample-size calculation was performed. Statistical analysis was performed using R [19]. Two-sided P-values are considered here and a P-value < 0.05 was considered significant.

## Results

**Fig. 1:**
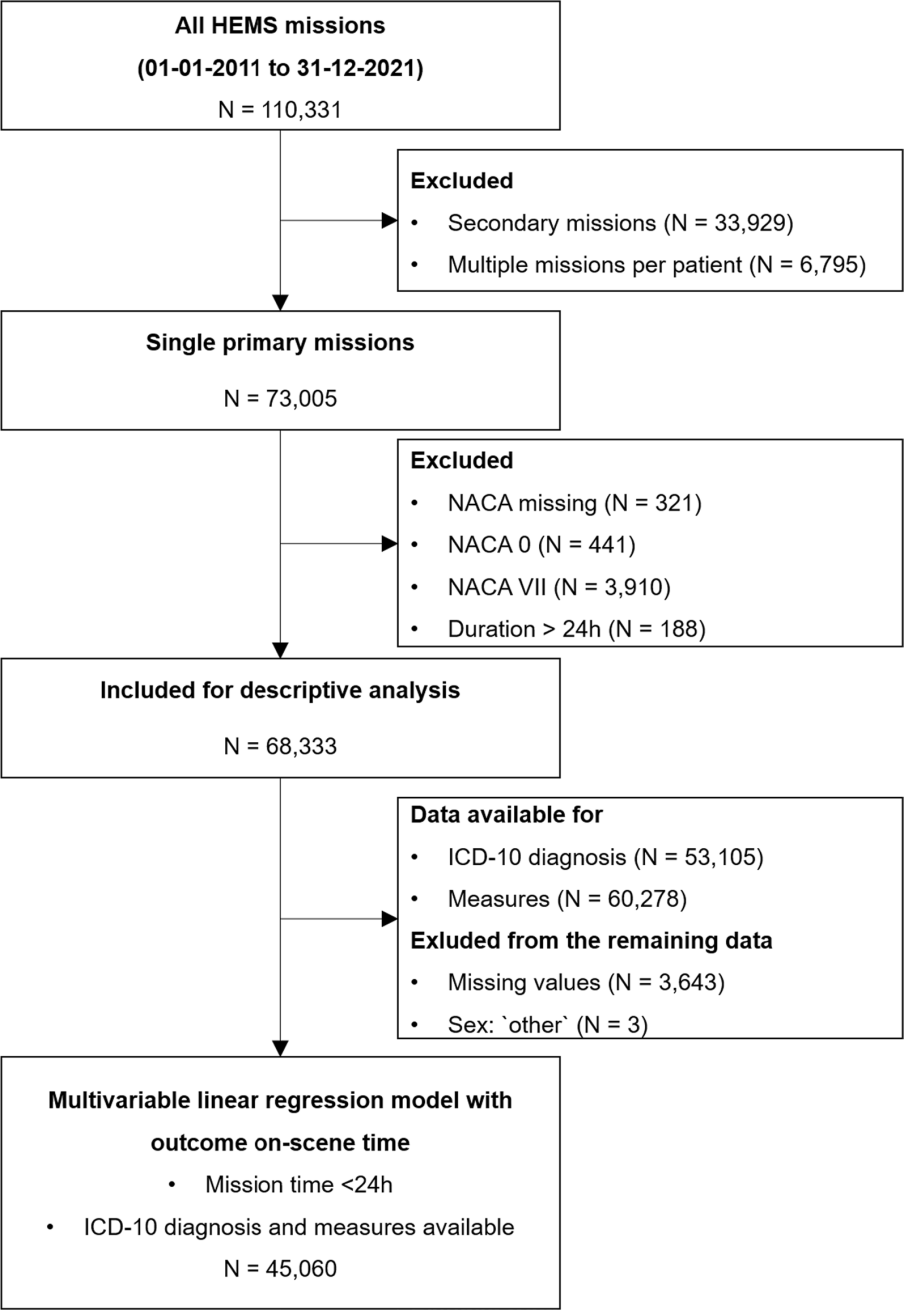
Study flowchart

We screened 110,331 missions, of which 68,333 were used in the final analysis. Figure 1 shows the study flow chart.

Patients and mission characteristics are summarised in Table 2. The median age was 50.0 [IQR: 27.0–65.0] years and most patients were adults (87.1%) and male (64.4%). The majority (52.4%) of all patients presented NACA scores between IV and VI. Overall, 11.0% of missions required a helicopter hoist operation (HHO), and the majority of missions took place during the daytime (86.1%). Across all included missions, prehospital time and on-scene time were 50.6 [IQR: 41.0;62.0] minutes and 21.0 [15.0;28.6] minutes, respectively.

**Table 2:** Baseline characteristics of all patients adjusted to age. Data are given in n (%) or median [Q1;Q3]

	All patients	Children (< 16 years)	Adults (≥ 16 years)	*p*	N
	*N* = *68,333*	*N* = *8,803*	*N* = *59,530*		
**Age** (years)	50.0 [27.0;65.0]	10.0 [5.00;13.0]	54.0 [36.0;68.0]	0.000	68,333
**Sex**:				< 0.001	68,333
Female	24,317 (35.6%)	3,628 (41.2%)	20,689 (34.8%)		
Male	44,010 (64.4%)	5,174 (58.8%)	38,836 (65.2%)		
Other	6 (0.01%)	1 (0.01%)	5 (0.01%)		
**National Advisory Committee for ﻿Aeronautics (NACA) score :**				< 0.001	68,333
NACA I–III	32,496 (47.6%)	5,396 (61.3%)	27,100 (45.5%)		
NACA IV–VI	35,837 (52.4%)	3,407 (38.7%)	32,430 (54.5%)		
**Glasgow Coma Scale** (GCS)**:**				< 0.001	64,918
3	5,106 (7.87%)	307 (3.64%)	4,799 (8.49%)		
4	335 (0.52%)	27 (0.32%)	308 (0.55%)		
5	318 (0.49%)	34 (0.40%)	284 (0.50%)		
6	558 (0.86%)	72 (0.85%)	486 (0.86%)		
7	523 (0.81%)	66 (0.78%)	457 (0.81%)		
8	519 (0.80%)	76 (0.90%)	443 (0.78%)		
9	505 (0.78%)	97 (1.15%)	408 (0.72%)		
10	780 (1.20%)	124 (1.47%)	656 (1.16%)		
11	869 (1.34%)	122 (1.45%)	747 (1.32%)		
12	938 (1.44%)	132 (1.57%)	806 (1.43%)		
13	1,892 (2.91%)	262 (3.11%)	1,630 (2.89%)		
14	6,238 (9.61%)	737 (8.75%)	5501 (9.74%)		
15	46,337 (71.4%)	6,369 (75.6%)	39,968 (70.7%)		
**Location**				< 0.001	62,158
Urban	32,283 (51.9%)	3,789 (46.4%)	28,494 (52.8%)		
Remote	29,875 (48.1%)	4,381 (53.6%)	25,494 (47.2%)		
**Helicopter hoist operation** (HHO):				< 0.001	68,333
No	60,785 (89.0%)	8,527 (96.9%)	52,258 (87.8%)		
Yes	7,548 (11.0%)	276 (3.14%)	7,272 (12.2%)		
**Activity:**				< 0.001	63,943
Traffic	9,301 (14.5%)	821 (9.5%)	8,480 (15.3%)		
Sport (Summer)	4,204 (6.6%)	399 (4.6%)	3,805 (6.9%)		
Sport Mountains (Summer)	5,727 (9.0%)	317 (3.7%)	5,410 (9.8%)		
Sport (Winter)	16,970 (26.5%)	3,977 (45.8%)	12,993 (23.5%)		
Sport Mountains (Winter)	1,197 (1.9%)	13 (0.15%)	1,184 (2.14%)		
Water Sports	579 (0.9%)	113 (1.30%)	466 (0.84%)		
Other	25,965 (40.6%)	3,046 (35.1%)	22,919 (41.5%)		
**Time of day:**				< 0.001	68,333
Daytime (07:00–19:00)	58,842 (86.1%)	7,818 (88.8%)	51,024 (85.7%)		
Night-time (otherwise)	9,491 (13.9%)	985 (11.2%)	8,506 (14.3%)		
**Prehospital time** (min)	50.6 [41.0;62.0]	47.0 [39.0;57.0]	51.0 [41.5;63.0]	< 0.001	64,899
**On-scene time** (min)	21.0 [15.0;28.6]	19.0 [14.0;25.0]	21.0 [15.2;29.0]	< 0.001	64,765

Detailed diagnoses, summarised in Table 3, were available in 53,105 missions (77.7% of all missions). Among these missions, 68.5% and 39.8% of patients were diagnosed with trauma and non-trauma, respectively. Significantly, Table 3 highlights that multiple trauma diagnoses were made in 39.3% of trauma patients and multiple non-trauma diagnoses in 32.9% of non-trauma patients. Note that in this analysis, we explicitly accounted for the possibility of both trauma and non-trauma diagnoses in a single patient.

**Table 3:** Diagnoses and on-scene times stratified by age. Data are given in n (%) or median [Q1;Q3]

	All patients	Children (< 16 years)	Adults (≥ 16 years)	*p*	N
	*N* = *68,333*	*N* = *8,803*	*N* = *59,530*		
**Diagnosis available:**	53,105 (77.7%)	6,889 (78.3%)	46,216 (77.6%)	0.195	68,333
*Trauma*					
**Trauma diagnosis**:	36,354 (68.5%)	5,647 (82.0%)	30,707 (66.4%)	< 0.001	53,105
**Number of trauma diagnoses:**				< 0.001	36,354
1	22,068 (60.7%)	3,779 (66.9%)	18,289 (59.6%)		
2	8,435 (23.2%)	1325 (23.5%)	7,110 (23.2%)		
3	4,152 (11.4%)	446 (7.90%)	3,706 (12.1%)		
4	1,699 (4.7%)	97 (1.7%)	1,602 (5.2%)		
**Prehospital time** (min)	49.0 [39.6;61.0]	46.0 [38.1;55.4]	50.0 [40.0;62.0]	< 0.001	34,811
**On-scene time** (min)	20.0 [15.0;28.0]	18.8 [14.0;24.0]	20.0 [15.0;28.3]	< 0.001	34,719
*Non-Trauma*					
**Non-trauma diagnosis**:	21,144 (39.8%)	1,618 (23.5%)	19,526 (42.2%)	< 0.001	53,105
**Number of non-trauma diagnoses:**				< 0.001	21,144
1	14,194 (67.1%)	1,380 (85.3%)	12,814 (65.6%)		
2	4,836 (22.9%)	211 (13.0%)	4,625 (23.7%)		
3	1,623 (7.68%)	25 (1.55%)	1,598 (8.18%)		
4	491 (2.32%)	2 (0.12%)	489 (2.50%)		
**Type of non-trauma:**					
Circulatory system	12,037 (56.9%)	199 (12.3%)	11,838 (60.6%)	< 0.001	21,144
Respiratory system	1398 (6.6%)	294 (18.2%)	1104 (5.7%)	< 0.001	21,144
Nervous system	2545 (12.0%)	342 (21.1%)	2203 (11.3%)	< 0.001	21,144
Other	6415 (30.3%)	805 (49.8%)	5610 (28.7%)	< 0.001	21,144
**Prehospital time** (min)	53.0 [44.0;64.0]	50.6 [42.0;60.0]	53.0 [44.0;64.0]	< 0.001	19,920
**On-scene time** (min)	22.0 [16.0;30.0]	21.0 [15.8;28.0]	22.0 [16.2;30.0]	< 0.001	19,922

Table 4 summarises the interventions and monitoring conducted, stratified into the following categories: basic interventions and monitoring, immobilisation and analgesia, critical interventions, airway management and resuscitation. Detailed variables were available in 60,278 (88.2%) missions. Intravenous access and SpO_2_ monitoring were established in 82.8% and 85.0% of missions, respectively. Analgesia was given in 50.3% of missions, while intraosseous access was performed in 0.9% of all missions where the measures were available. As regards airway management, tracheal intubation and capnography were performed in 10.3% and 11.4% of all missions, respectively. Chest compressions were performed as a resuscitation measure in 2.5% of all missions. Significantly, in 91.5% of all missions with recorded measures, further measures, as mentioned in Table 1, were taken beyond those explicitly mentioned in Table 4. While most patients were immobilised with the vacuum mattress, cervical collar and vacuum splinting were rarely used.

**Table 4:** Available measures of interventions and monitoring stratified by age Data are given in n (%)

	All patients	Children (< 16 years)	Adults (≥ 16 years)	p	N
	*N* = *68,333*	*N* = *8,803*	*N* = *59,530*		
**Measure available:**	60,278 (88.2%)	7,923 (90.0%)	52,355 (87.9%)	< 0.001	68,333
*Basic interventions and monitoring*					
iv access	49,923 (82.8%)	4,761 (60.1%)	45,162 (86.3%)	< 0.001	60,278
SpO_2_	51,214 (85.0%)	6,353 (80.2%)	44,861 (85.7%)	< 0.001	60,278
ECG	30,974 (51.4%)	2,038 (25.7%)	28,936 (55.3%)	< 0.001	60,278
Temperature	3,073 (5.10%)	421 (5.31%)	2,652 (5.07%)	0.363	60,278
*Immobilisation & analgesia*					
Vacuum mattress	24,555 (40.7%)	4,041 (51.0%)	20,514 (39.2%)	< 0.001	60,278
Sedation	613 (1.0%)	130 (1.6%)	483 (0.9%)	< 0.001	60,278
Analgesia	31,928 (53.0%)	3,627 (45.8%)	28,301 (54.1%)	< 0.001	60,278
Reduction of a fracture or dislocation	1,048 (1.7%)	124 (1.6%)	924 (1.8%)	0.222	60,278
Haemostasis	1,460 (2.4%)	104 (1.3%)	1,356 (2.6%)	< 0.001	60,278
*Critical interventions*					
i.o. access	548 (0.9%)	110 (1.4%)	438 (0.8%)	< 0.001	60,278
eFONA	14 (0.02%)	1 (0.01%)	13 (0.02%)	> 0.99	60,278
Chest needle decompression	182 (0.3%)	8 (0.1%)	174 (0.3%)	0.001	60,278
transcutaneous paceing	139 (0.2%)	1 (0.01%)	138 (0.3%)	< 0.001	60,278
Vasopressors	3,544 (5.9%)	202 (2.6%)	3342 (6.4%)	< 0.001	60,278
*Airway management*					
Tracheal intubation	6,210 (10.3%)	421 (5.3%)	5,789 (11.1%)	< 0.001	60,278
Facemask ventilation	2,822 (4.7%)	265 (3.3%)	2,557 (4.9%)	< 0.001	60,278
NMBA	3,834 (6.4%)	251 (3.2%)	3,583 (6.8%)	< 0.001	60,278
Capnography	6,884 (11.4%)	473 (6.0%)	6,411 (12.2%)	< 0.001	60,278
*Resuscitation*					
Chest compressions	1,476 (2.5%)	104 (1.3%)	1,372 (2.6%)	< 0.001	60,278
Defibrillation	8,68 (1.4%)	22 (0.3%)	846 (1.6%)	< 0.001	60,278
Mechanical chest compression device	1,128 (1.9%)	26 (0.3%)	1,102 (2.1%)	< 0.001	60,278
*Other*					
Other*	55,131 (91.5%)	6,727 (84.9%)	48,404 (92.5%)	< 0.001	60,278

*ECG* electrocardiogram, *eFONA* emergency front of neck access, *iv* intravenous, *i.o.* intraosseous, *NMBA* neuromuscular blocking agent, *SpO*_*2*_ peripheral oxygen saturation

*Including but not limited to: wound dressing, vacuum splinting and cervical collar

**Fig. 2:**
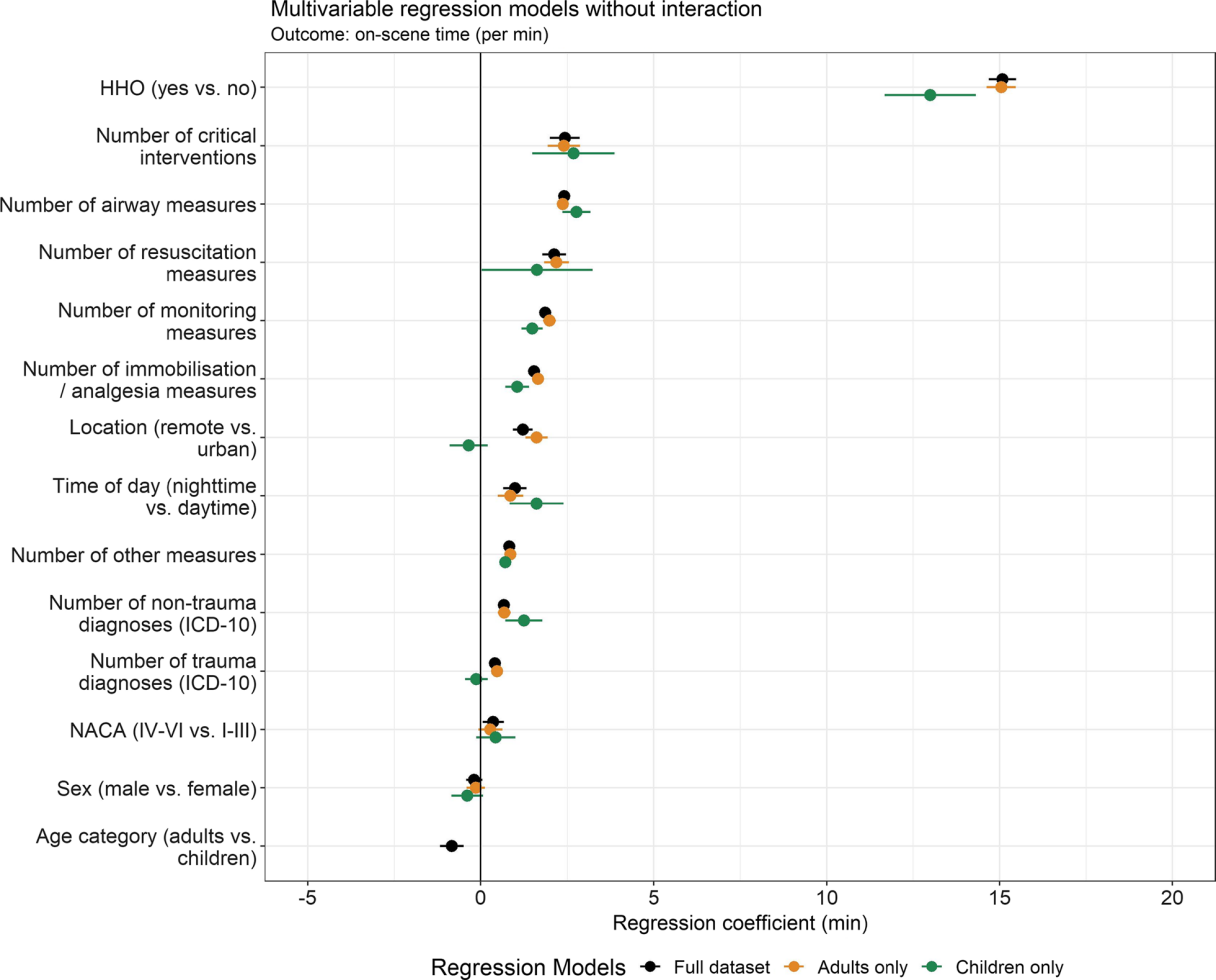
Regression coefficients of the multivariable linear regression model with ‘on-scene time’ as the outcome. Mean and 95% confidence intervals are shown. Separate regression models were computed for (i) all patients, (ii) the child sub-group and (iii) the adult sub-group. *HHO* helicopter hoist operations, *NACA* National Advisory Committee for Aeronautics

Data including ICD-10 diagnosis, interventions and monitoring to be entered into the multivariable regression model with the outcome ‘on-scene time’ were available for 45,060 missions (paediatric n = 5,981, adults n = 39,079), as summarised in Table 5. In the model, helicopter hoist operations (HHO) had the largest effect on on-scene time: a mission requiring HHO adds on average 15.1 (95%-CI 14.7–15.5, p < 0.001) minutes to the overall on-scene time. Table 5 highlights that each critical intervention and each airway-management measure adds on average 2.4 (95%-CI 2.0–2.9, p < 0.001) minutes and 2.4 (95%-CI 2.3–3.6, p < 0.001) minutes, respectively. Note that separate regression models were computed for the child and adult sub-groups. To aid the interpretation and relative size of the regression coefficient, Fig. 2 illustrates each variable’s estimates of the regression coefficients (in units of minutes) in decreasing order.

**Table 5:** The regression coefficient of multivariable linear regression with the outcome on-scene time (in minutes) stratified by age

Outcome: On-scene time (min)	All patients			Children (< 16 years)			Adults (16 ≥ years)		
Characteristic	Beta	95% CI	p	Beta	95% CI	p	Beta	95% CI	p
**Helicopter hoist operation (HHO)**									
No	–	–		–	–		–	–	
Yes	15.1	14.7, 15.5	< 0.001	13.0	11.7, 14.3	< 0.001	15.1	14.6, 15.5	< 0.001
**Critical interventions** (per measure)	2.4	2.0, 2.9	< 0.001	2.7	1.5, 3.9	< 0.001	2.4	1.9, 2.9	< 0.001
**Airway management** (per measure)	2.4	2.3, 2.6	< 0.001	2.8	2.4, 3.2	< 0.001	2.4	2.2, 2.5	< 0.001
**Resuscitation** (per measure)	2.1	1.8, 2.5	< 0.001	1.6	0.03, 3.2	0.046	2.2	1.8, 2.5	< 0.001
**Basic interventions and monitoring** (per measure)	1.9	1.7, 2.0	< 0.001	1.5	1.2, 1.8	< 0.001	2.0	1.8, 2.2	< 0.001
**Immobilisation and analgesia** (per measure)	1.5	1.4, 1.7	< 0.001	1.1	0.72, 1.4	< 0.001	1.7	1.5, 1.8	< 0.001
**Location**									
Urban	–	–		–	–		–	–	
Remote	1.2	0.93, 1.5	< 0.001	− 0.35	− 0.90, 0.20	0.2	1.6	1.3, 1.9	< 0.001
**Time of day**									
Daytime (07:00–19:00)	–	–		–	–		–	–	
Night-time (otherwise)	1.0	0.65, 1.3	< 0.001	1.6	0.84, 2.4	< 0.001	0.86	0.49, 1.2	< 0.001
Other measures* (per measure)	0.82	0.75, 0.90	< 0.001	0.71	0.54, 0.87	< 0.001	0.85	0.77, 0.93	< 0.001
**Non-trauma** (per diagnosis)	0.67	0.49, 0.84	< 0.001	1.2	0.71, 1.8	< 0.001	0.68	0.49, 0.86	< 0.001
**Trauma** (per diagnosis)	0.41	0.28, 0.55	< 0.001	− 0.13	− 0.45, 0.20	0.4	0.47	0.32, 0.62	< 0.001
**National Advisory Committee for Aeronautics (NACA) score**									
NACA I–III	–	–		–	–		–	–	
NACA IV–VI	0.36	0.05, 0.67	0.021	0.43	− 0.13, 1.0	0.13	0.28	− 0.07, 0.62	0.12
**Sex**									
Female	–	–		–	–		–	–	
Male	− 0.19	− 0.42, 0.05	0.12	− 0.39	− 0.85, 0.07	0.094	− 0.14	− 0.41, 0.12	0.3
**Age category**									
Children	–	–		–	–		–	–	
Adults	− 0.83	− 1.2, − 0.49	< 0.001						
*Model performance*									
**Number of observations** (N)	45,060			5,981			39,079		
**Adjusted R-squared**	0.23			0.26			0.23		

*including but not limited to: wound dressing, vacuum splinting and cervical collar

**Fig. 3:**
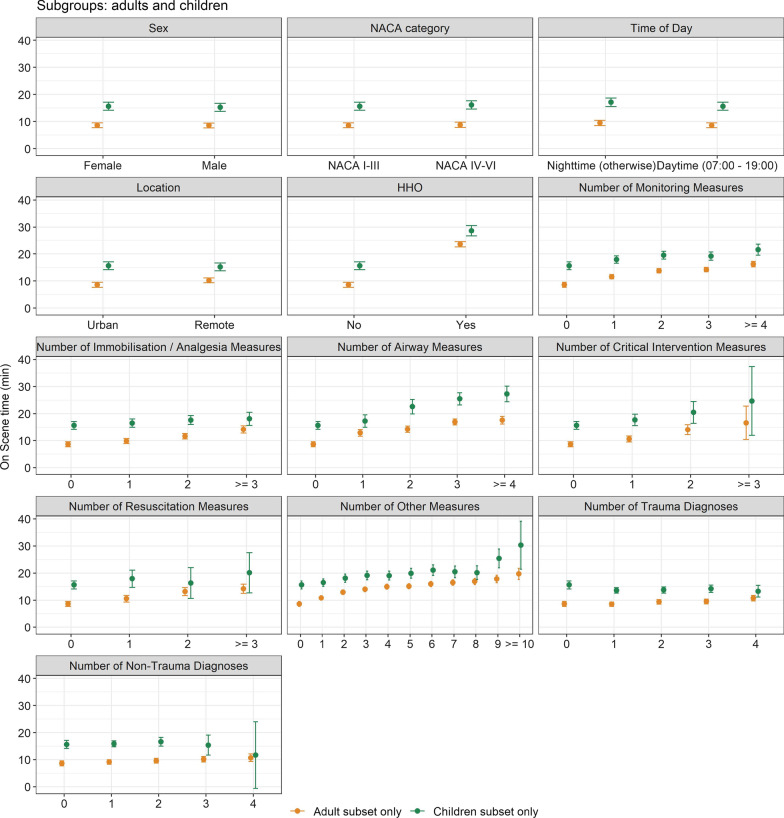
Effect plots of the multivariable linear regression model with the outcome ‘on-scene time’ (in minutes), separately for the child and adult sub-groups. Mean and 95% confidence intervals are shown. Note that the number of diagnoses and measures are considered as factor variables here to assess the linearity of the association between the number of measures and on-scene time. *HHO* helicopter hoist operations, *NACA* National Advisory Committee for Aeronautics

This study explicitly considers the number and types of measures taken (e.g. airway management measures): the impact of multiple measures on on-scene time in the child and adult sub-groups is shown in Fig. 3, which shows that the on-scene time increases linearly as a function of the number of airway-management, monitoring and critical-intervention measures.

## Discussion

In summary, in Swiss Air-Rescue’s physician-staffed HEMS, adjusted on-scene and prehospital time for missions with paediatric patients was slightly longer than in missions with adult patients. Patients in remote areas that are difficult to access due to the terrain, requiring an evacuation involving a helicopter hoist, were associated with the greatest prolongation of on-scene time. Critical interventions, resuscitation and airway management also influenced the on-scene time to a varying degree (Fig. 3). Every single intervention and monitoring measure has a cumulative effect on on-scene time.

An observational study reported median HEMS on-scene times of only 10 min, which may be explained by a high proportion of treatment interventions being conducted by ground-based medical teams before final helicopter transport [20]. A German comparative registry analysis of trauma patients between 2007 and 2009 showed on-scene times of around 40 min for HEMS and explained these in terms of a high proportion of patients requiring airway management [21]. Patient survival for HEMS transport was improved in this German analysis compared to the cohort of ground transportation by ambulance. In our analysis, on-scene time significantly depended on the interventions and measurements performed. It remains unclear whether—and if so to what extent—prolonged on-scene time in general influences patient outcomes [3]. Fixating solely on shortening on-scene times might not capture the reality for patients, as life-saving interventions performed at the prehospital stage substantially reduced mortality in trauma patients [7, 10]. Discovering the correct diagnosis may reduce the delivery time for definitive treatment. For example, stroke patients had improved functional outcomes when treated by a mobile prehospital stroke unit as compared to traditional treatment in hospital [22].

Helicopters enable missions to rescue patients, especially in remote and difficult terrain. Given the topography of Switzerland, with the Alps extending up to 4,634 m and many narrow valleys, these patients might be impossible to reach by ground ambulances. Many missions in this analysis involved patients undertaking recreational activities in the mountains in summer and winter (e.g. skiing, hiking or climbing). Rescue missions with ground-based evacuation teams could, to the patient’s disadvantage, easily require several hours just to reach the patient. A HEMS helicopter operating with a helicopter hoist can bring a physician to the patient directly and in a timely way, resulting in immediate access to clinical investigations and interventions [23]. This could be crucial, as an analysis of helicopter hoist missions in a physician-staffed HEMS reported that nearly 20% of the patients were severely injured and presented with a NACA score ≥ 4 [6]. Such helicopter hoist operations can avoid lengthy terrestrial evacuations, which potentially endanger both patients and rescue crews. Although our data revealed that helicopter hoist operations were associated with the greatest prolongation of on-scene time, the additional on-scene time spent in these HEMS missions results in substantially less rescue time and likely greater chances of neurologically intact survival, as compared to traditional ground rescue.

Advanced airway management in a patient with respiratory failure or the need for a patent airway is a potentially life-saving intervention, which is performed safely by physician-staffed HEMS services, often anaesthesiologists [24]. This skill could even be performed in-cabin as an en-route treatment to optimise time management during the flight to the hospital [25]. Resuscitation was associated with prolonged on-scene time. Performing cardiopulmonary resuscitation in the cabin by HEMS is challenging due to the helicopter’s limited personnel resources and working space. Nonetheless, mechanical chest compression devices facilitate high-quality cardiopulmonary resuscitation in such situations [26]. No evidence, such as outcome data, is yet available for cases after the use of mechanical resuscitation devices under HEMS conditions. Unfortunately, most of these devices cannot be used with paediatric patients, as reflected by the low numbers in our cohort.

Critical interventions were regularly performed and associated with prolonged on-scene time. Most common in this cohort was the administration of intravenous vasopressors to maintain perfusion, intraosseous access and chest needle decompression.

Intravenous access and analgesia were less frequently reported in children than in adults, even though children had more trauma. Our findings are in line with a recent observational study in a physician-staffed HEMS [27]. Even if missions involving children were rare and physicians might not be that thoroughly trained in paediatric rescue, not to mention afraid of the potential risks, the reasons behind this inequity are unclear and require further investigation. Furthermore, the adjusted model revealed a slightly longer on-scene time for paediatric patients, while unadjusted on-scene time was shorter. This might be explained by statistical confounding, as paediatric patients had lower NACA scores compared to adults. However, while this finding was statistically significant, the clinical importance of a prolonged on-scene time of only 1 min might be negligible.

Modifiable variables, such as the duration of a single measure (e.g. a critical intervention), have a significantly larger impact on on-scene time than non-modifiable factors, such as age category, type and number of diagnoses, and NACA score. Thus, reducing the duration of a *single* measure (intervention or monitoring) in these categories or performing these measures *in parallel* or during the flight as in-cabin treatment may hold significant potential to reduce on-scene time.

While the measurements in our model were considered statistically independent, several measurements affect each other in clinical practice: A patient with a cardiac arrest is likely to be treated at least with chest compressions, defibrillation, intravenous or intraosseous access, vasopressors, intubation and capnography. Considering the time for all the single measurements, a cardiopulmonary resuscitation might easily last 10–15 min on-scene. For patients requiring emergency anaesthesia, baseline monitoring (SpO2, electrocardiogram, non-invasive blood pressure, and capnography), intravenous or intraosseous access, several medications (e.g. hypnotic, opioid, neuromuscular blocking agent, vasopressors), facemask ventilation, intubation and mechanical ventilation might also result in at least 10–15 min spent on-scene. In our HEMS, one medical crew member is responsible for preparing and administering medication and hemodynamic monitoring, while the other oversees airway management. These parallel performed tasks might save time spent on-scene. However, communication is important in such situations to have shared mental models within the HEMS crew; thus, an airway checklist is performed as standard operating procedure [28].

Our study has several limitations due to its retrospective and observational character. Data in some mission reports (i.e. ICD-10 diagnosis or measurement) are missing. The time of arrival on-scene is the landing time of the helicopter. Thus, the on-scene time might be slightly overestimated*.* We considered patients with a NACA score ≥ IV potentially time-critical. However, only the patient's most severe NACA score was recorded, which might be resolved by early on-scene treatment (e.g. airway obstruction, tension pneumothorax, anaphylactic shock). Our data may be difficult to compare with those from other topographic areas with less need for helicopter hoist operations. Unfortunately, our database lacks patient-survival and outcome data, which we realised is an important point for improvement on this study. Finally, the study took place over a long period of time which might have had influence in terms of protocols and practice.

## Conclusions

In conclusion, compared to adult patients, the adjusted on-scene and the prehospital time for children was slightly longer, and children were more likely to have trauma, but also a lower NACA score. Intravenous access and analgesia were less frequently established in children. On-scene time was significantly prolonged in rescue missions with helicopter hoist operations. Each individual intervention and monitoring measure increases on-scene time. Thus, performing such interventions in parallel or as in-cabin treatment could be an option to reduce on-scene time in life-threatening patient conditions, but would require special training. However, multiple clinical interventions and monitoring interact and are not single interventions. Compared to the impact of interventions, non-modifiable factors, such as NACA score, type of diagnosis and age, make only a minor contribution to overall on-scene time. Future research should focus on the crucial association between on-scene time and patient outcomes in a physician-staffed HEMS, quality of care for paediatric prehospital patients and the feasibility of in-cabin treatment.

## Data Availability

The dataset analysed in the current study is available from the corresponding author upon reasonable request and with permission of the responsible Ethics Committee.

## Abbreviations

HEMS: Helicopter Emergency Medical Service
HHO: Helicopter Hoist Operation
ICD: International Classification of Diseases
NACA: National Advisory Committee for Aeronautics

## References

[1] Oude Alink MB, Moors XRJ, Karrar S, Houmes RJ, Hartog DD, Stolker RJ. Characteristics, management and outcome of prehospital pediatric emergencies by a Dutch HEMS. Eur J Trauma Emerg Surg. 2021; 10.1007/s00068-020-01579-8 PMC9001565 33543366

[2] Mueller S, Zheng J, Orav EJ, Schnipper JL (2019). Inter-hospital transfer and patient outcomes: a retrospective cohort study. BMJ Qual Saf.

[3] Spoelder EJ, Slagt C, Scheffer GJ, van Geffen GJ (2022). Transport of the patient with trauma: a narrative review. Anaesthesia.

[4] Fuchs A, Schmucki R, Meuli L, Wendel-Garcia PD, Albrecht R, Greif R (2022). Helicopter inter-hospital transfer for patients undergoing extracorporeal membrane oxygenation: a retrospective 12-year analysis of a service system. Scand J Trauma Resuscit Emerg Med.

[5] Meuli L, Zimmermann A, Menges AL, Tissi M, Becker S, Albrecht R (2021). Helicopter emergency medical service for time critical interfacility transfers of patients with cardiovascular emergencies. Scand J Trauma Resuscit Emerg Med.

[6] Pietsch U, Knapp J, Mann M, Meuli L, Lischke V, Tissi M (2021). Incidence and challenges of helicopter emergency medical service (HEMS) rescue missions with helicopter hoist operations: analysis of 11,228 daytime and nighttime missions in Switzerland. Scand J Trauma Resuscit Emerg Med.

[7] Gomes E, Araujo R, Carneiro A, Dias C, Costa-Pereira A, Lecky FE (2010). The importance of pre-trauma centre treatment of life-threatening events on the mortality of patients transferred with severe trauma. Resuscitation.

[8] Berkeveld E, Popal Z, Schober P, Zuidema WP, Bloemers FW, Giannakopoulos GF (2021). Prehospital time and mortality in polytrauma patients: a retrospective analysis. BMC Emerg Med.

[9] Gauss T, Ageron FX, Devaud ML, Debaty G, Travers S, Garrigue D (2019). Association of prehospital time to in-hospital trauma mortality in a physician-staffed emergency medicine system. J Am Med Assoc Surg.

[10] Brown JB, Rosengart MR, Forsythe RM, Reynolds BR, Gestring ML, Hallinan WM (2016). Not all prehospital time is equal: influence of scene time on mortality. J Trauma Acute Care Surg.

[11] Blasius FM, Horst K, Brokmann JC, Lefering R, Andruszkow H, Hildebrand F, et al. Helicopter emergency medical service and hospital treatment levels affect survival in pediatric trauma patients. J Clin Med. 2021;10(4). 10.3390/jcm10040837 PMC7922049 33670679

[12] Englum BR, Rialon KL, Kim J, Shapiro ML, Scarborough JE, Rice HE (2017). Current use and outcomes of helicopter transport in pediatric trauma: a review of 18,291 transports. J Pediatr Surg.

[13] Knapp J, Haske D, Bottiger BW, Limacher A, Stalder O, Schmid A (2019). Influence of prehospital physician presence on survival after severe trauma: systematic review and meta-analysis. J Trauma Acute Care Surg.

[14] Li T, Cushman JT, Shah MN, Kelly AG, Rich DQ, Jones CMC (2021). Prehospital time intervals and management of ischemic stroke patients. Am J Emerg Med.

[15] Herlitz J, Wireklintsundstrom B, Bang A, Berglund A, Svensson L, Blomstrand C (2010). Early identification and delay to treatment in myocardial infarction and stroke: differences and similarities. Scand J Trauma Resuscit Emerg Med.

[16] Liu VX, Fielding-Singh V, Greene JD, Baker JM, Iwashyna TJ, Bhattacharya J (2017). The timing of early antibiotics and hospital mortality in sepsis. Am J Respir Crit Care Med.

[17] von Elm E, Altman DG, Egger M, Pocock SJ, Gøtzsche PC, Vandenbroucke JP (2007). The Strengthening the Reporting of Observational Studies in Epidemiology (STROBE) statement: guidelines for reporting observational studies. Lancet.

[18] World Health Organization (WHO). International Classification of Diseases (ICD-10) 2019 https://icd.who.int/browse10/2019/en#/U07.12019. https://icd.who.int/browse10/2019/en#/U07.1.

[19] R Core Team. R: A language and environment for statistical computing. R Foundation for Statistical Computing, Vienna, Austria. 2021.

[20] Osteras O, Heltne JK, Vikenes BC, Assmus J, Brattebo G (2017). Factors influencing on-scene time in a rural Norwegian helicopter emergency medical service: a retrospective observational study. Scand J Trauma Resuscit Emerg Med.

[21] Andruszkow H, Lefering R, Frink M, Mommsen P, Zeckey C, Rahe K (2013). Survival benefit of helicopter emergency medical services compared to ground emergency medical services in traumatized patients. Crit Care.

[22] Ebinger M, Siegerink B, Kunz A, Wendt M, Weber JE, Schwabauer E (2021). Association between dispatch of mobile stroke units and functional outcomes among patients with acute ischemic stroke in Berlin. J Am Med Assoc.

[23] Ausserer J, Moritz E, Stroehle M, Brugger H, Strapazzon G, Rauch S (2017). Physician staffed helicopter emergency medical systems can provide advanced trauma life support in mountainous and remote areas. Injury.

[24] Pietsch U, Mullner R, Theiler L, Wenzel V, Meuli L, Knapp J (2022). Airway management in a Helicopter Emergency Medical Service (HEMS): a retrospective observational study of 365 out-of-hospital intubations. BMC Emerg Med.

[25] Knapp J, Venetz P, Pietsch U (2021). In-cabin rapid sequence induction : experience from alpine air rescue on reduction of the prehospital time. Anaesthesist.

[26] Pietsch U, Reiser D, Wenzel V, Knapp J, Tissi M, Theiler L (2020). Mechanical chest compression devices in the helicopter emergency medical service in Switzerland. Scand J Trauma Resuscit Emerg Med.

[27] Rugg C, Woyke S, Ausserer J, Voelckel W, Paal P, Strohle M (2021). Analgesia in pediatric trauma patients in physician-staffed Austrian helicopter rescue: a 12-year registry analysis. Scand J Trauma Resuscit Emerg Med.

[28] Fuchs A, Frick S, Huber M, Riva T, Theiler L, Kleine-Brueggeney M, et al. Five-year audit of adherence to an anaesthesia pre-induction checklist. Anaesthesia. 2022. 10.1111/anae.15704 PMC9314793 35302235

